# Moderate phosphorus reduction under split drip fertigation maintains maize yield and coincides with closer root–available phosphorus spatial correspondence

**DOI:** 10.3389/fpls.2026.1999323

**Published:** 2026-10-13

**Authors:** Changle Liu, Shuping Hu, Daling Ma, Liqing Wang, Xiaofang Yu, Julin Gao

**Affiliations:** 1 College of Agronomy, Inner Mongolia Agricultural University, Hohhot, China; 2 Maize Research Institute, Inner Mongolia Academy of Agricultural & Animal Husbandry Sciences, Hohhot, China

**Keywords:** Olsen P, phosphorus fertigation, phosphorus use efficiency, root length density, spatial coordination, spring maize

## Abstract

**Introduction:**

Phosphorus (P) has low mobility in soil, so crop P acquisition depends on both P availability and its spatial accessibility to roots. We hypothesized that moderate P reduction under split fertigation could maintain maize productivity by improving the coordination between available P and root distribution.

**Methods:**

A two-year field experiment was conducted in irrigated spring maize in Inner Mongolia, China. Split monoammonium phosphate fertigation was applied at 105 (CP1), 127.5 (CP2), and 150 kg P_2_O_5_ ha^-1^ (CP3), together with a no-P treatment (CP0) and conventional basal diammonium phosphate application at 150 kg P_2_O_5_ ha^-1^ (CK). Root length density and Olsen P were measured in paired soil blocks within the 0–40 cm soil profile at silking, together with plant P accumulation, biomass, grain yield, and P-use efficiency.

**Results:**

CP2 increased Olsen P availability and root proliferation in the middle and deeper soil layers and showed closer spatial correspondence between root length density and Olsen P at 20–40 cm. These changes were associated with greater post-silking P accumulation and biomass. Reducing P input by 15% maintained grain yield at the level of CP3 and increased yield by 4.07% relative to CK, while apparent P recovery efficiency and P agronomic efficiency increased by 77.36% and 54.62%, respectively. Further reducing P input to 105 kg P_2_O_5_ ha⁻¹ decreased P accumulation, biomass, and yield, whereas 150 kg P_2_O_5_ ha^-1^ provided no additional yield benefit.

**Discussion:**

Under the conditions of this study, moderate P reduction under split fertigation maintained maize yield while reducing fertilizer input and enhancing P-use efficiency, and this response was associated with closer spatial correspondence between root distribution and soil available P.

## 1 Introduction

Phosphorus (P) is essential for crop growth and development because of its central roles in energy transfer, nucleic acid and membrane synthesis, metabolic regulation, root development, and reproductive processes (Suriyagoda et al., 2023; Yang et al., 2023). However, efficient P management remains a major challenge in crop production. Phosphate has low mobility in soil and is readily sorbed or precipitated after fertilizer application, such that only a limited proportion of applied P is recovered by crops during the season of application (Tiecher et al., 2023; Li et al., 2024b). Repeated high P inputs have consequently generated substantial residual or legacy P pools in agricultural soils and catchments, increasing the risk of P transfer to aquatic systems, while phosphate rock itself is a finite and unevenly distributed resource (Alewell et al., 2020; He et al., 2023). Recent evidence suggests that fertilizer P use in China could be substantially reduced through management based on soil P status without compromising crop production (Gong et al., 2025). Thus, a central challenge for sustainable P management is not simply to increase soil P availability, but to improve the efficiency with which crop roots encounter and acquire available P so that grain yield can be maintained with lower fertilizer inputs.

This challenge is particularly relevant to the irrigated spring maize region of northern China. In Inner Mongolia, spring maize is produced over a large area under intensive water and fertilizer management and contributes substantially to regional and national grain production (He et al., 2018; Zhou et al., 2025a). In conventional crop production, P fertilizer is often applied as a single basal dose at or before sowing, with little or no additional P supplied during the subsequent growing season (He et al., 2024). To minimize the risk of P deficiency, however, fertilizer rates often exceed crop demand. Because of the low mobility of phosphate, a large proportion of basal P remains concentrated in the upper soil layer (Li et al., 2024a; McDowell et al., 2024), whereas the maize root system progressively expands into deeper soil during crop development (Gao et al., 2024). Consequently, the accumulation of P near the soil surface may create a spatial mismatch between soil P availability and root distribution as maize roots increasingly explore deeper soil layers during the middle and late growth stages (Chen et al., 2022; Guo et al., 2024). Increasing P input does not necessarily overcome this mismatch; instead, excessive application can accelerate soil P accumulation, decrease fertilizer P use efficiency, and increase the risk of P loss to surrounding water bodies (Xu et al., 2023; Chen et al., 2025). Improving the spatiotemporal coordination between available P and root distribution may therefore be more important than simply increasing the amount of P applied.

Drip fertigation provides a potential means of improving this coordination because water and nutrients can be supplied repeatedly and directly to the crop root zone (Ma et al., 2022; Guo et al., 2023). However, P fertigation differs fundamentally from fertigation with mobile nutrients such as nitrate N. Phosphorus moves mainly by diffusion over short distances and is rapidly adsorbed or precipitated after entering the soil (Meyer et al., 2023; McKenna et al., 2024). Therefore, the agronomic benefit of split P fertigation is unlikely to depend simply on transporting P over long distances with irrigation water (Wang et al., 2024a). Rather, repeated delivery of P within the wetted root zone may alter the location and persistence of available P patches and thereby influence root foraging (Jing et al., 2022; Zhang et al., 2023a). Because maize roots are morphologically plastic, local variation in soil nutrient availability can alter root proliferation, while localized P enrichment can increase root growth in P rich zones and enlarge the contact interface between roots and soil (Lambers, 2022; Yin et al., 2024). Thus, the effectiveness of split P fertigation may depend on whether it creates a spatial distribution of available P that coincides with the developing root system. From this perspective, crop P acquisition depends not only on how much available P is present in the soil, but also on where that P is located relative to active roots.

Previous studies have shown that split P fertigation, fertilizer source, placement method, and dripline burial depth can affect soil Olsen P distribution, root growth, plant P uptake, and crop yield (Liu et al., 2023; Wang et al., 2024a; Yamini and Singh, 2024). Nevertheless, two important questions remain unresolved for drip irrigated spring maize. First, most previous studies have evaluated P sources, placement strategies, or dripline configurations at a fixed P rate, leaving uncertain how far fertilizer P inputs can be reduced under a given split fertigation regime without constraining plant P accumulation or grain yield (Yan et al., 2021; Freiling et al., 2022). Second, many previous studies have characterized soil available P and root distribution separately or at the scale of predefined soil layers, making it difficult to determine whether root proliferation and relatively high P availability actually coincide within the same local soil zones (Liu et al., 2022; Li et al., 2025). This distinction is important because increases in total root length or soil Olsen-P alone do not necessarily improve P acquisition if roots and available P remain spatially separated (Bilyera et al., 2022; Holz et al., 2024). It therefore remains unclear whether moderate P reduction under split fertigation can maintain available P in the middle and deeper portions of the root zone, promote root proliferation in these same soil domains, and thereby sustain P acquisition and crop productivity.

Here, we conducted a field experiment over two years in irrigated spring maize in Inner Mongolia to determine how P application rate under split fertigation affects the vertical distribution of soil Olsen P and maize roots, their local spatial association, plant P accumulation, grain yield, and P use efficiency. Split monoammonium phosphate fertigation at three P rates was compared with a no P treatment and the locally conventional one time basal diammonium phosphate application. Root length density and Olsen P were quantified in paired soil blocks within the 0–40 cm profile at silking, enabling their spatial association to be evaluated at a finer spatial scale than is possible with conventional measurements averaged across soil layers. We hypothesized that moderately reducing P input under split fertigation would improve the spatial coordination between available P and root distribution in the middle and deeper root zone, thereby sustaining P acquisition and grain yield while increasing P use efficiency. Specifically, we aimed to determine (i) how P rate under split fertigation alters the vertical distributions of available P and maize roots, (ii) whether these changes improve local root-P spatial concordance, and (iii) which P rate provides the best balance between crop productivity and fertilizer P use efficiency.

## 2 Materials and methods

### 2.1 Experimental site

Field experiments were conducted during the 2022 and 2023 growing seasons at the China Chilechuan Modern Agricultural Expo Park, Tumoteyou Qi, Baotou, Inner Mongolia, China (40°28′28″N, 110°29′5″E; 1015 m). The site is located on the Tumochuan Plain and has a temperate continental semiarid monsoon climate, with approximately 140 d without frost. The soil was a sandy loam, with initial total N, available N, available K, available P, organic matter, and pH of 1.54 g kg^-1^, 74.89 mg kg^-1^, 136.28 mg kg^-1^, 15.23 mg kg^-1^, 17.83 g kg^-1^, and 7.98 in 2022. Seasonal precipitation from May to September was 652.3 mm in 2022 and 445.86 mm in 2023, and the corresponding effective accumulated temperatures were 1887 °C and 1921 °C. Rainfall, irrigation events, fertigation timing, and crop phenology are shown in **Figure 1**.

**Figure 1:**
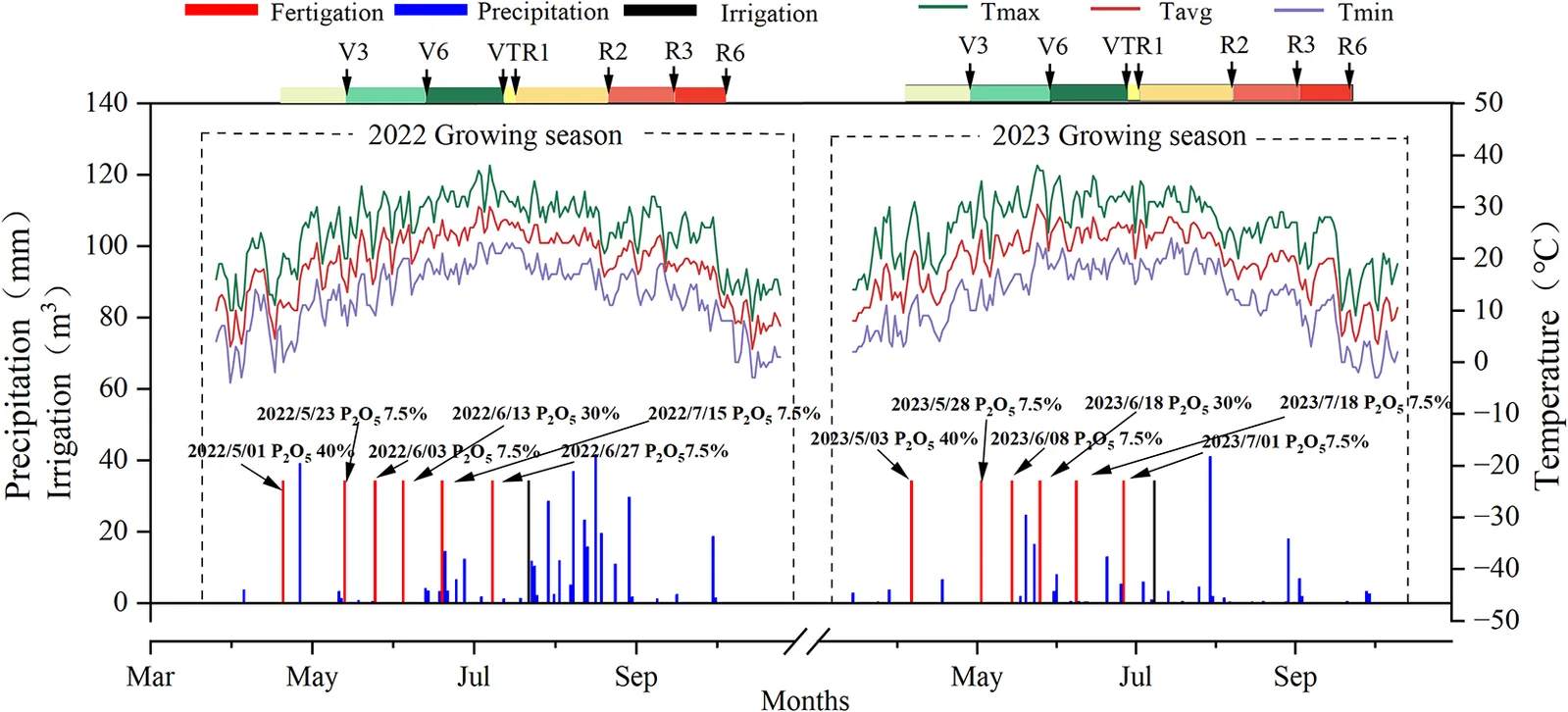
Key meteorological variables during the maize growing season in the experimental area.

### 2.2 Experimental design

A randomized block design was used in this experiment. Five treatments were set: no phosphorus application (CP0, 0 kg P_2_O_5_ ha^-1^), split phosphorus application with a 30% reduction (CP1, 105 kg P_2_O_5_ ha^-1^), split phosphorus application with a 15% reduction (CP2, 127.5 kg P_2_O_5_ ha^-1^), split phosphorus application at the conventional phosphorus rate (CP3, 150 kg P_2_O_5_ ha^-1^), conventional basal phosphorus application at sowing (CK, 150 kg P_2_O_5_ ha^-1^). There were 5 treatments with 3 replicates, giving a total of 15 plots. Each plot had an area of 90 m². In treatments CP1, CP2, and CP3, monoammonium phosphate (MAP; 60% P_2_O_5_ and 12% N) was applied with irrigation water through the drip irrigation system. In contrast, in the CK treatment, diammonium phosphate (DAP; 46.4% P_2_O_5_ and 18% N) was applied once as a basal fertilizer at sowing. To account for differences in nutrient composition between the two P fertilizer sources and to maintain consistent total N and K_2_O inputs among treatments, additional N and K fertilizers were supplied during split MAP application. Urea containing 46% N and potassium chloride containing 62% K_2_O were used as the supplemental N and K sources, respectively. Across all treatments, the total N application rate was 300 kg N ha^-1^, and the total K_2_O application rate was 105 kg K_2_O ha^-1^.

All plots were irrigated using the same drip irrigation system with shallow buried drip lines. In CP1-CP3, MAP was applied at sowing, V6, V8, V12, R1, and R2. During each fertigation event, the fertilizer valve was opened for 120 min and then flushed with clean water for 180 min to improve nutrient distribution and reduce emitter clogging. Irrigation amount and timing were identical among treatments. The P application schedules are shown in **Tables 1**, **2** and **Figure 2**. The same plots were used in both years.

**Table 1:** Phosphorus application treatments and application methods.

Code	Treatment	Fertilization method
CP0	No P application under drip irrigation (100% reduction in the locally recommended P rate)	No P fertilization
CP1	Split P fertigation with a 30% reduction in the locally recommended P rate	Split fertigation
CP2	Split P fertigation with a 15% reduction in the locally recommended P rate	Split fertigation
CP3	Split P fertigation at the locally recommended P rate	Split fertigation
CK	Conventional basal P application at sowing with tillage	Basal fertilization at sowing

The irrigation volume and number of irrigations were consistent across the treatments CK, CP0, CP1, CP2, and CP3.

**Table 2:** Phosphorus application schedule and distribution ratio under different treatments.

Treatment	Total P input (kg P_2_O_5_ ha^-1^)	Basal P fertilizer (kg P_2_O_5_ ha^-1^)	P application through drip irrigation (kg P_2_O_5_ ha^-1^)					
			Sowing	V6	V8	V12	R1	R2
CP0	0	0	0	0	0	0	0	0
CP1	105.0	0	42.000	7.875	7.875	31.500	7.875	7.875
CP2	127.5	0	51.000	9.563	9.563	38.250	9.563	9.563
CP3	150.0	0	60.000	11.250	11.250	45.000	11.250	11.250
CK	150.0	150.0	0	0	0	0	0	0

**Figure 2:**
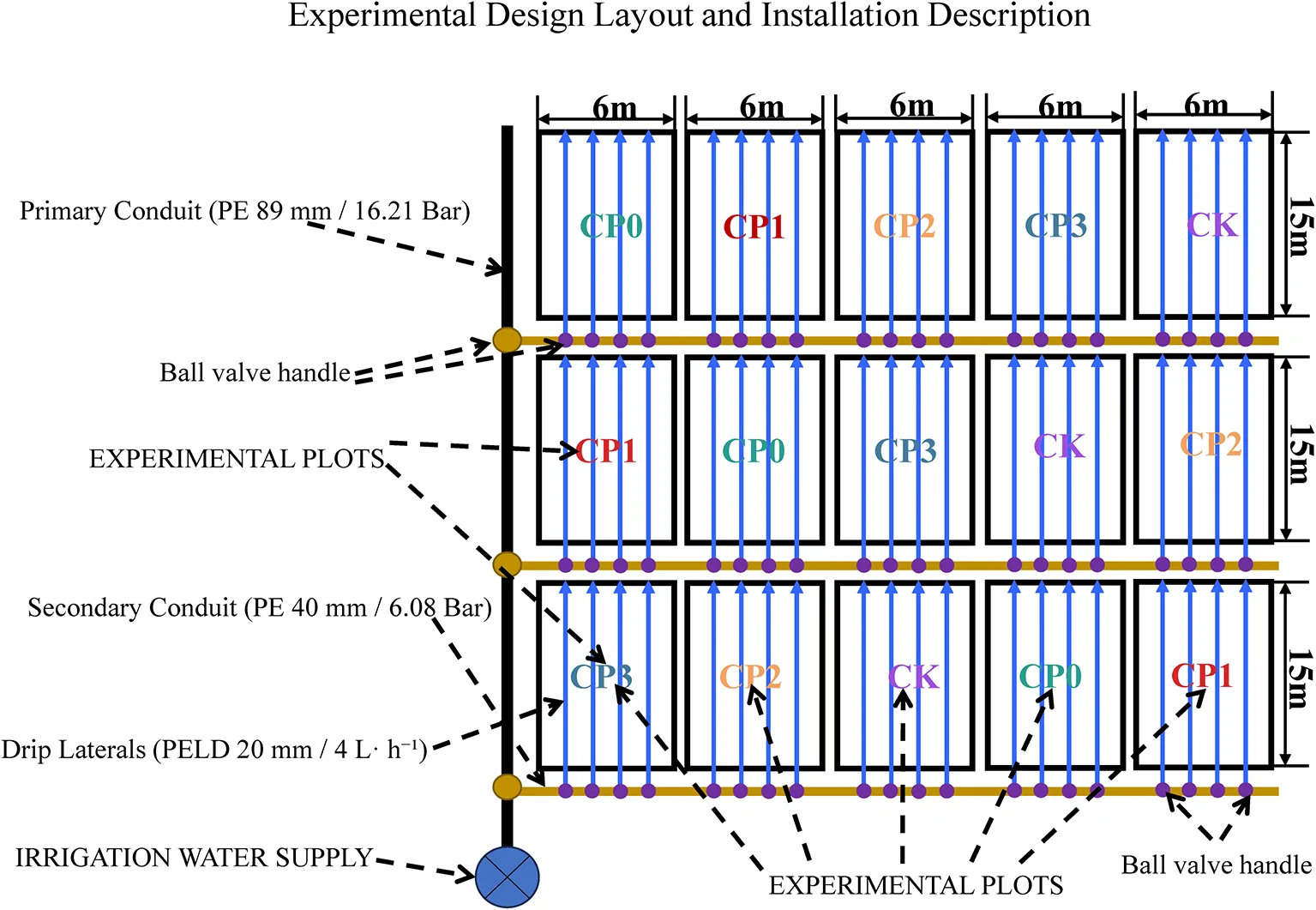
Experimental plot layout and drip irrigation system configuration.

Maize (cultivar Dika 159) was sown on 28 April 2022 and 30 April 2023 at a target density of 10.5 × 10^4^ plants ha^-1^. The crop was irrigated using a subsurface drip irrigation system with inlaid drip lines buried at a depth of 2–3 cm below the soil surface (diameter: 20 mm; wall thickness: 0.2 mm; emitter spacing: 10 cm; nominal discharge rate: 4 L h^-1^). From seedling emergence to harvest, irrigation was applied every 7–15 days, totaling seven irrigation events and a seasonal irrigation amount of 3600 m^3^ ha^-1^. Each irrigation event delivered an equal amount of water to all plots. Irrigation and fertigation were managed independently using separate water and fertilizer tanks, with application rates precisely controlled by water meters, fertigation tanks, and valves to ensure treatment consistency. All plots received identical irrigation volumes and schedules. Other field management practices, including pest and disease control and weed management, were conducted following local agronomic recommendations and were kept consistent across all treatments to ensure experimental uniformity.

Conventional urea [CO(NH_2_)_2_; 46% N] was used as the N fertilizer, and potassium chloride (KCl; 62% K_2_O) was used as the K fertilizer. The P fertilizers differed between application methods: monoammonium phosphate (MAP; NH_4_H_2_PO_4_; 12% N and 60% P_2_O_5_) was applied in split applications through drip irrigation, whereas diammonium phosphate (DAP; 18% N and 46.4% P_2_O_5_) was applied once as a basal fertilizer and incorporated into the soil by rotary tillage at maize sowing. The experimental layout and drip irrigation configuration are shown in **Figure 2**.

### 2.3 Measurements and methods

#### 2.3.1 Aboveground biomass

At R1 and R6, three representative maize plants were sampled from each plot and separated into leaves, stems (including tassels, bracts, cobs, and ear shanks), and grains. The samples were heated at 105 °C for 30 min and subsequently oven dried at 80 °C to constant weight. The dry weight of each organ was recorded, and total dry matter accumulation per plant was calculated (Zhang et al., 2023b).

#### 2.3.2 Yield

At physiological maturity, maize ears from each plot were harvested to determine grain yield under different phosphorus treatments. To minimize border effects, two central rows with a total length of 10 m were selected from each plot for yield determination. The harvested ears were dried in air to a constant weight and mechanically threshed. Grain moisture content was measured using a digital grain moisture meter (Model LDS-5H, Kehui, China), and grain weight was adjusted to a standard moisture content of 14%. The plot yield (kg m^-2^) was then converted to grain yield per hectare (t ha^-1^) based on the harvested area. For each treatment, the mean value across replications was used for statistical analysis (Cheng et al., 2025).

#### 2.3.3 Root measurements

At the silking stage of maize (65–70 d after sowing), one representative plant was randomly selected from each replicate plot for root profile sampling, resulting in three plants per treatment. Root sampling time followed (Sha et al., 2025). According to the “3D monolith” method for root spatial sampling (Ding et al., 2021), the maize plant was taken as the center, and the X axis was set parallel to the planting row, the Y axis was set perpendicular to the planting row, and the Z axis represented soil depth. Each sampling unit had a volume of 10 cm × 10 cm × 10 cm. Soil samples were collected layer by layer from 0 to 40 cm depth. A total of 36 soil samples were collected for each plant, with 9 samples in each layer, for root measurement and analysis of soil Olsen P, as shown in **Supplementary Figure S1**. The root samples were scanned using an Epson Perfection V700 Photo scanner, and root length, root surface area (RSA), root volume (RV), and root dry weight (RDW) were determined using the WinRHIZO 2016 root analysis system; root length density was then calculated. After scanning, the roots were dried in an oven to constant weight and weighed for dry matter. where RLD is root length density (cm cm^-3^), L is root length (cm), and V is soil volume (cm³).

Root length density (RLD) was calculated as RLD = L/V, where L is root length (cm) and V is the soil block volume (cm^3^). The depth containing 95% of cumulative root length was defined as D95. D95 was estimated by interpolation using an exponential model, following (Hund et al., 2009).

#### 2.3.4 Soil Olsen P content

Soil samples were collected at the same positions as the roots. At the plants used for root profile sampling, 36 soil samples were collected from the 0–40 cm soil profile using a 3 × 3 × 4 grid of 10 × 10 × 10 cm³, and each soil sample corresponded to one root sample. The samples were taken back to the laboratory, dried in air naturally, cleaned of visible plant and animal residues, passed through a 2 mm sieve, and then ground. Olsen P was extracted by the Olsen method using 0.5 mol L^-1^ NaHCO_3_ (pH 8.5) at a soil:solution ratio of 1:20. The suspension was shaken at 25 °C for 30 min and then filtered. Olsen P in the filtrate was determined using a colorimetric method based on molybdenum and antimony with a Smartchem 450 automatic chemical analyzer (AMS, France).

#### 2.3.5 Plant phosphorus accumulation and calculation of phosphorus fertilizer use efficiency

At V12, R1 and R6, three representative plants were collected from the center of each plot. Plants were separated into leaves, stems (including tassels, bracts, cobs, and ear shanks), and grains. Samples were heated at 105 °C for 30 min, dried at 80 °C to constant weight, weighed, and ground. Total P concentration was determined after H_2_SO_4_-H_2_O_2_ digestion using a Smartchem 450 automatic chemical analyzer (AMS, France).

The phosphorus fertilizer use efficiency indices were calculated as follows (Chen et al., 2024).

Plant P accumulation was calculated as follows:


$$\text{Plant P accumulation }\left({\text{kg ha}}^{-1}\right)=\text{dry weight }\times \text{ P concentration}\times \text{ plant density.}$$

P accumulation after silking was calculated as:


$$\text{P accumulation after silking }\left({\text{kg ha}}^{-1}\right)=\text{total aboveground P accumulation at R}6-\text{aboveground P accumulation at R}1,$$

where total aboveground P accumulation at R6 was the sum of P accumulation in vegetative organs and grain at R6.

Phosphorus recovery efficiency was calculated as:


$$\text{PRE}\left(\%\right)=\left({\text{PA}}_{\text{n}}-{\text{PA}}_{\text{o}}\right)/{\text{FP}}_{\text{n}}\times 100.$$

Phosphorus agronomic efficiency was calculated as:


$$\text{PAE}\left({\text{kg kg}}^{-1}\right)=\left({\text{GY}}_{\text{n}}-{\text{GY}}_{\text{o}}\right)/{\text{FP}}_{\text{n}}.$$

Partial factor productivity of phosphorus was calculated as:


$$\text{PFPP}\left({\text{kg kg}}^{-1}\right)={\text{GY}}_{\text{n}}/{\text{FP}}_{\text{n}},$$

where GY_o_ is the grain yield of the no P treatment (CP0), and GY_n_ is the grain yield of a treatment receiving P fertilizer. PA_o_ is the total aboveground P accumulation of CP0, and PA_n_ is the total aboveground P accumulation of a treatment receiving P fertilizer. FP_n_ is the applied phosphorus fertilizer rate expressed as P_2_O_5_ (kg P_2_O_5_ ha^-1^). In this study, n represents CP1, CP2, CP3, and CK.

#### 2.3.6 Descriptive correlation analysis between RLD and Olsen P

To describe the local spatial association between root distribution and soil available P, paired RLD and Olsen P values were obtained from nine corresponding soil blocks within each plot and soil layer. The 27 paired observations from the three replicate plots were pooled within each treatment, year, and soil layer to visualize the relationship between RLD and Olsen P using linear regression. As a sensitivity analysis accounting for the hierarchical sampling structure, linear regressions between RLD and Olsen P were also fitted separately within each replicate plot using the nine paired spatial observations, and the direction and magnitude of the resulting replicate-specific slopes were compared with those obtained from the pooled analysis.

### 2.4 Statistical analysis

Data were organized using Excel 2021. Before further analysis, the raw data for each variable (or model residuals) were tested for normality using the Kolmogorov Smirnov test and for homogeneity of variance using Levene’s test. For treatment comparisons of root traits and Olsen P within each soil layer, the nine spatial observations within each plot were averaged to obtain one plot-level value. The three replicate plots were then treated as independent experimental units (n = 3).Each plot was treated as one statistical unit, with three replicates for each treatment in each year. Statistical analysis was performed using SPSS 18.0 (IBM SPSS Statistics, USA). When the analysis of variance showed a significant effect, the least significant difference (LSD) test was used for multiple comparisons at the 0.05 level. Figures were prepared using Origin 2022 (OriginLab Corp., Northampton, MA, USA) and Surfer 11.0 (Golden Software, USA). The partial least squares structural equation model (PLS-SEM) was built in R version 4.1.3 using the plspm package.

## 3 Results

### 3.1 Profile distribution of soil Olsen P at the silking stage under split phosphorus application through drip irrigation

**Supplementary Table S1** shows that P treatment affected the vertical distribution of Olsen P in the 0–40 cm soil profile at silking. In the 0–10 cm layer, Olsen P did not differ significantly among CP2, CP3, and CK, but these treatments were higher than CP1 by 33.29%, 26.16%, and 23.64%, respectively. In the 10–20 cm layer, CK had significantly higher Olsen P than CP1, CP2, and CP3 by 44.80%, 49.02%, and 38.75%, respectively. By contrast, in the 20–30 cm layer, CP2 and CP3 had significantly higher Olsen P than CK by 19.37% and 34.58%, respectively. In the 30–40 cm layer, CP2 exceeded CK by 49.37% (*p < 0.05*). Compared with CK, CP2 and CP3 maintained higher Olsen P in the 20–30 cm soil layer, while CP2 also maintained higher Olsen P in the 30–40 cm layer (**Figure 3**).

**Figure 3:**
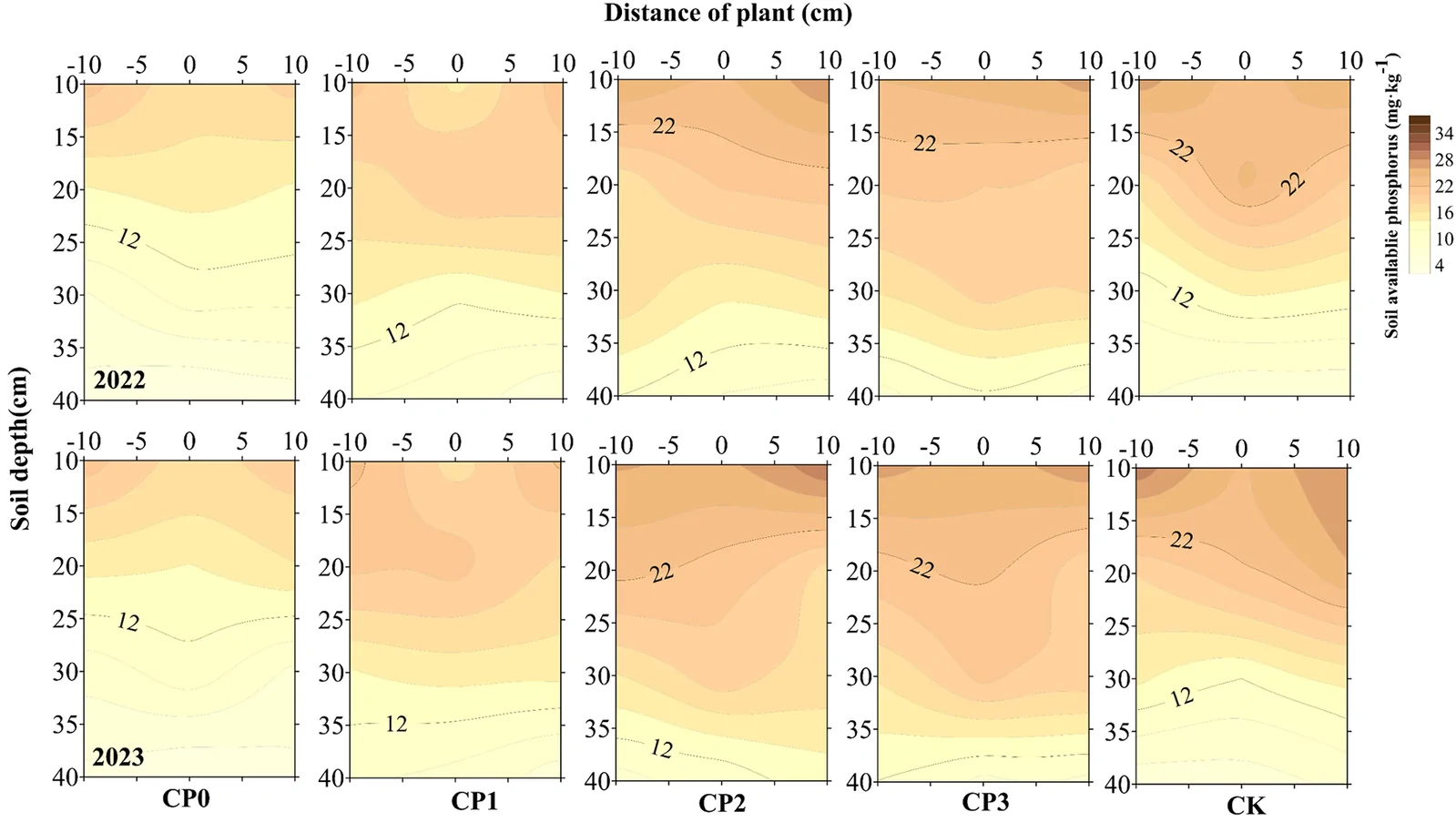
Vertical distribution of soil Olsen P in the 0–40 cm soil profile at the silking stage under different P treatments in 2022 (upper row) and 2023 (lower row).

### 3.2 Profile distribution of root morphological traits under split phosphorus application through drip irrigation

**Figure 4** shows that split P fertigation increased maize root growth in deeper soil layers and altered the vertical distribution of root morphological traits. In the 0–10 cm layer, RV and RSA did not differ among CP2, CP3, and CK. However, compared with CP1, CP2, CP3, and CK significantly increased RV by 17.14%, 13.39%, and 12.64%, respectively, and RSA by 10.69%, 9.35%, and 6.49%, respectively.

**Figure 4:**
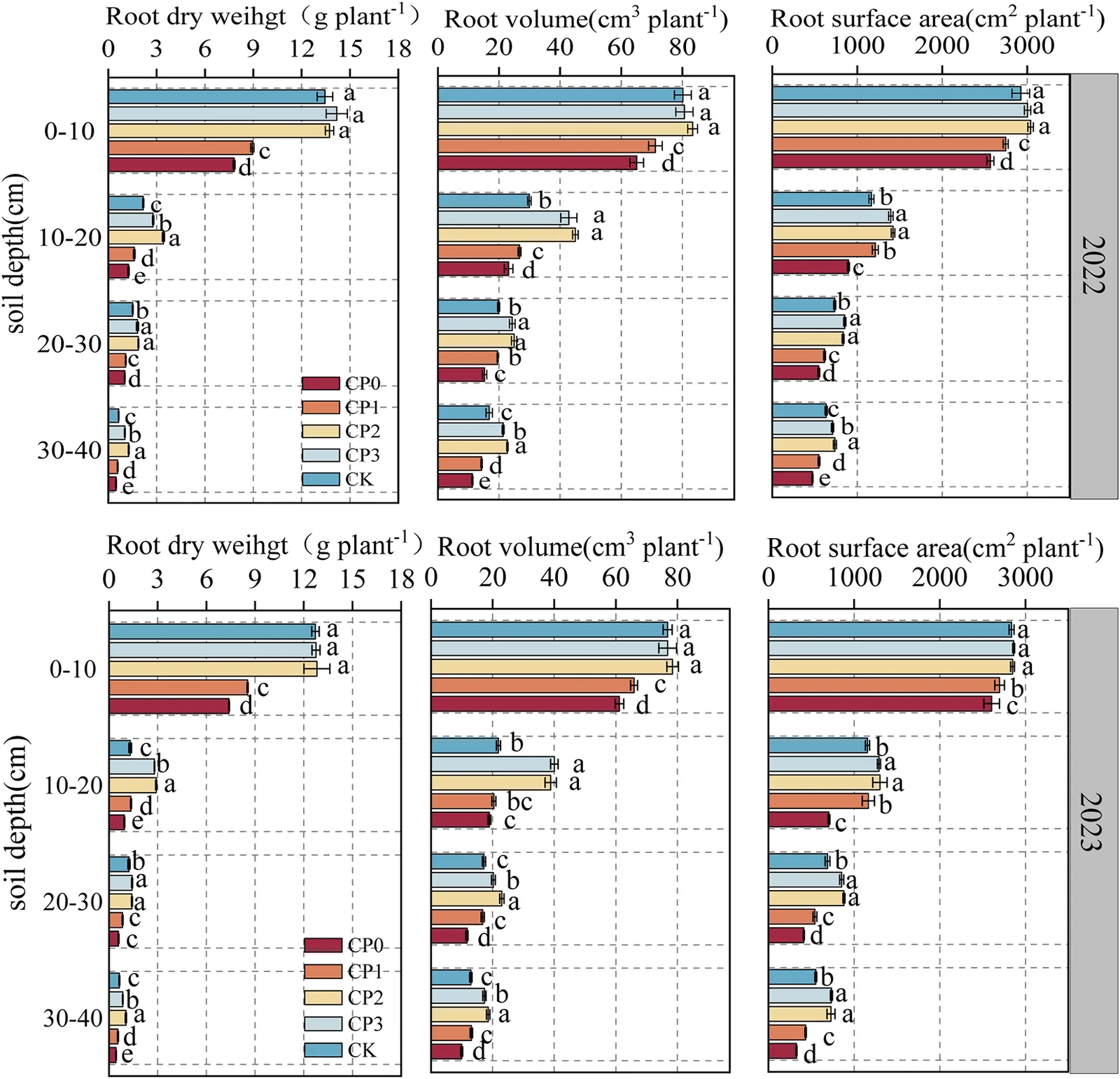
Effects of different phosphorus treatments on RDW, RV, and RSA in different soil layers at the silking stage of spring maize (2022 and 2023). Different lowercase letters indicate significant differences among treatments at p < 0.05.

In the 10–20 cm layer, RV and RSA did not differ between CP2 and CP3. Compared with CK, CP2 significantly increased RV and RSA by 50.28% and 21.72%, respectively, whereas CP3 increased them by 43.11% and 19.45%, respectively (*p < 0.05*). In the 20–30 cm layer, CP2 and CP3 also had similar RV and RSA, and both exceeded CK and CP1 (*p < 0.05*). In the 30–40 cm layer, CP2 had the greatest RV and RSA, exceeding CK by 35.61% and 16.21%, respectively (*p < 0.05*).

Overall, CP3 mainly promoted RV and RSA in the 10–30 cm layers, whereas CP2 extended the response to the 30–40 cm layer.

RDW showed a similar vertical response (**Figure 4**). In the 0–10 cm layer, RDW did not differ among CP2, CP3, and CK. In the 10–20 cm layer, CP2 had higher RDW than CP1, CP3, and CK by 113.37%, 13.32%, and 81.97%, respectively (*p < 0.05*). In the 20–30 cm layer, CP2 and CP3 did not differ from each other, but both were higher than CK and CP1 (*p<0.05*). In the 30–40 cm layer, CP2 again had the highest RDW, exceeding CP1, CP3, and CK by 103.26%, 22.74%, and 81.01%, respectively (*p < 0.05*).

**Figure 5** shows that split P fertigation increased RLD in deeper soil layers within the 0–40 cm profile. The D95 analysis indicated that vertical root distribution depth under CP3 was 6.14% greater than under CK in 2023 (*p < 0.05*) (**Figure 6**). Overall, CP2 and CP3 promoted root growth toward deeper soil layers.

**Figure 5:**
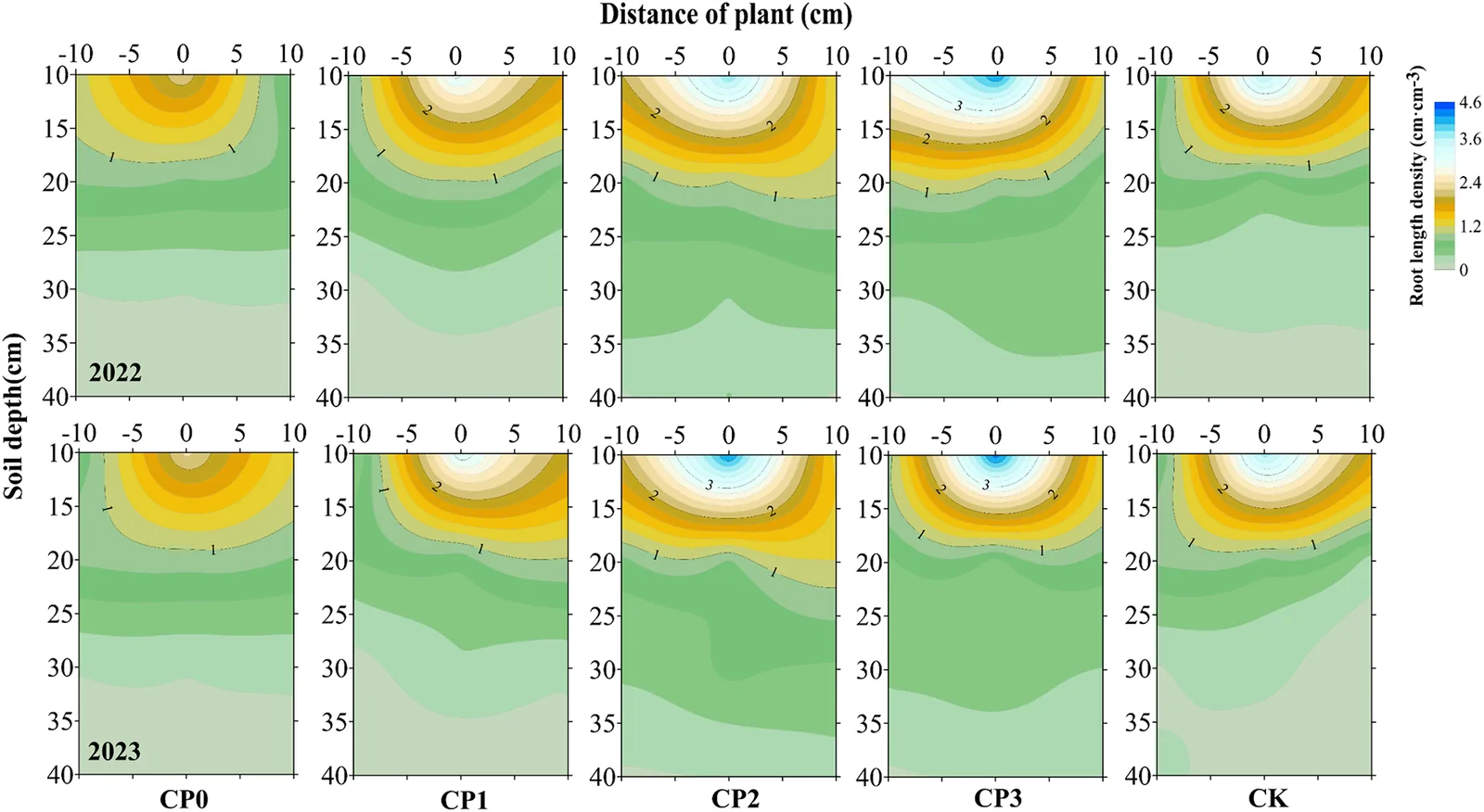
Vertical distribution of RLD in the 0–40 cm soil profile at the silking stage of spring maize under different phosphorus treatments in 2022 (upper row) and 2023 (lower row).

**Figure 6:**
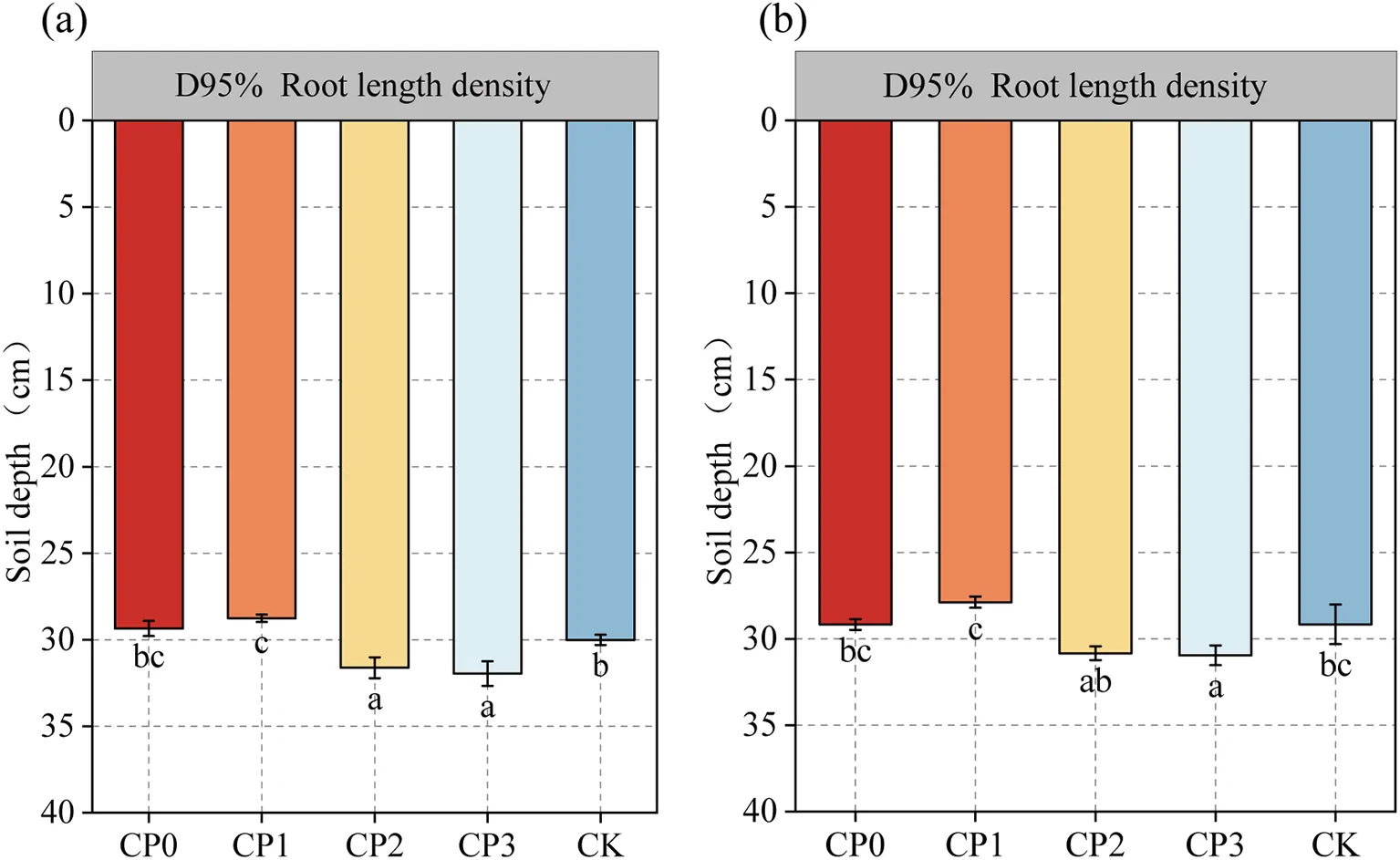
D95 values of root length density (RLD) in the 0–40 cm soil profile at the silking stage of spring maize under different phosphorus treatments in 2022 **(a)** and 2023 **(b)**. D95 indicates the depth at which 95% of cumulative root length was reached. Values are means ± SE (n = 3). Different lowercase letters indicate significant differences among treatments within each year at (*p<0.05*).

### 3.3 Analysis of the spatial matching between maize roots and phosphorus under split phosphorus application through drip irrigation

Under the CP2 treatment, root length density showed significant positive relationships with Olsen P contents in the 20–30 cm and 30–40 cm soil layers in both 2022 and 2023 (**Figures 7c, d, g, h**). A sensitivity analysis based on regressions fitted separately within each replicate plot showed consistently positive slopes across all three replicate plots in these two soil layers in both years. Thus, the positive RLD-Olsen P pattern under CP2 was robust to accounting for the hierarchical sampling structure.

**Figure 7:**
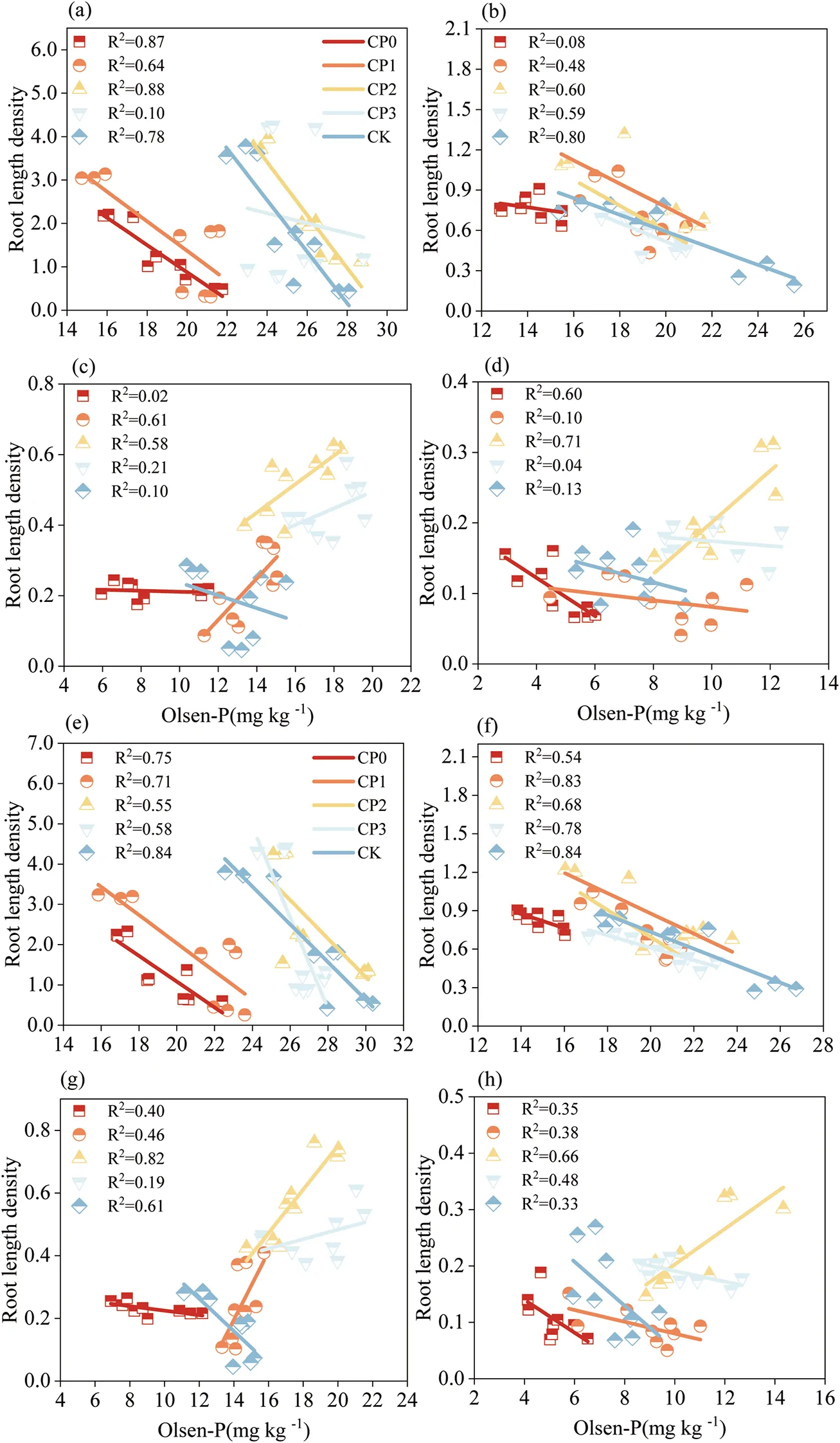
Descriptive relationships between root length density (RLD) and soil Olsen P in different soil layers at the silking stage of spring maize under different phosphorus treatments. Panels **(a/e, b/f, c/g, d/h)** correspond to the 0-10, 10-20, 20-30, and 30–40 cm soil layers, respectively. Points represent paired RLD and Olsen P observations from corresponding soil blocks. Panels **(a–d)** represent data from 2022, while panels **(e–h)** represent data from 2023.

### 3.4 Effects of split phosphorus application through drip irrigation on aboveground biomass of maize

**Figure 8** shows that CP2 and CP3 generally maintained greater aboveground biomass than CK and CP1, particularly at R6. At R1, biomass under CP2 was significantly higher than under CK and CP1 by 9.31% and 19.29%, respectively (*p < 0.05*). At R6, CP2 and CP3 did not differ from each other; however, CP2 increased biomass by 4.77% and 7.97% relative to CK and CP1, respectively, and CP3 increased biomass by 3.91% and 7.08%, respectively (*p < 0.05*). These results indicate that CP2 improved biomass accumulation before and after silking, whereas CP3 mainly increased biomass accumulation after silking.

**Figure 8:**
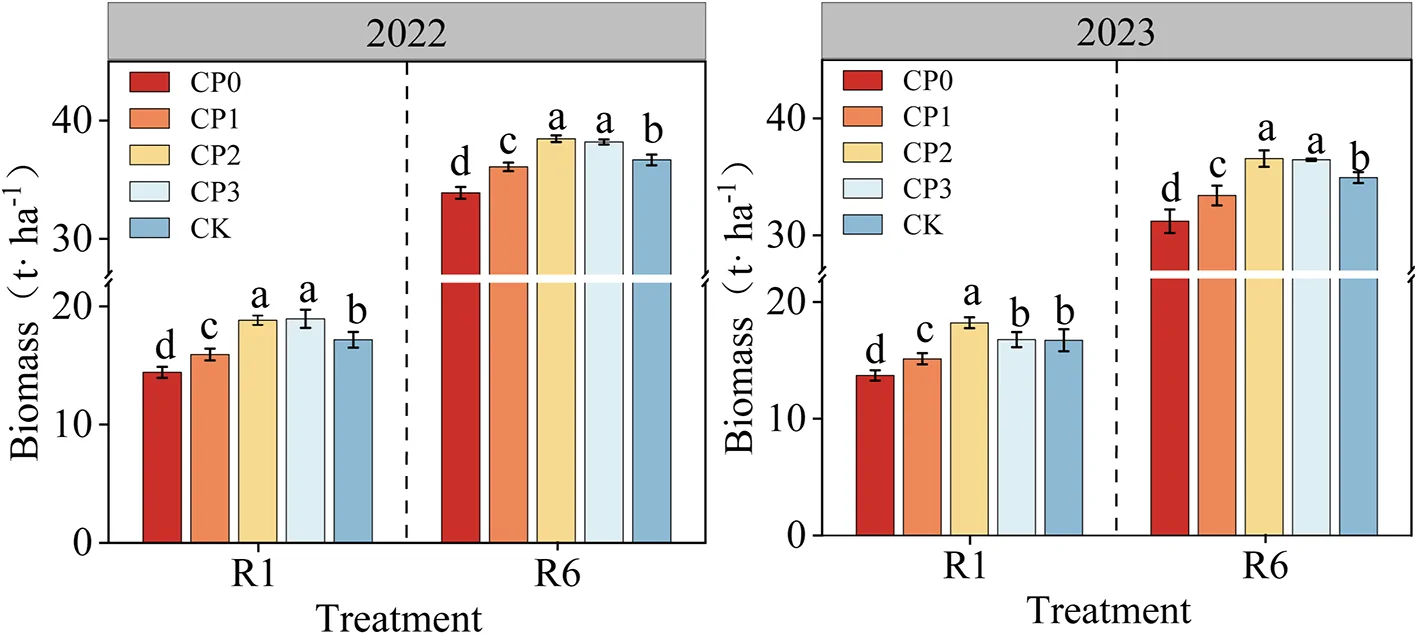
Effects of different phosphorus treatments on aboveground biomass of spring maize (2022 and 2023). Different lowercase letters indicate significant differences among treatments at p < 0.05.

### 3.5 Effects of split phosphorus application through drip irrigation on plant phosphorus accumulation and translocation

**Table 3** shows significant treatment differences in shoot P accumulation and grain P accumulation at V12, R1, and R6 (*p < 0.01*). Shoot P accumulation peaked at R1, with the order CP2 > CP3 > CK > CP1. At R6, CP2 and CP3 did not differ in shoot or grain P accumulation, but both were significantly higher than CP1 and CK (*p < 0.05*). Grain P accumulation under CP2 was 17.50% and 7.69% higher than under CP1 and CK, respectively (*p < 0.05*). P accumulation after silking under CP2 and CP3 did not differ significantly, but both exceeded CK by 9.85% and 9.46%, respectively (*p < 0.05*). These results indicate that split P fertigation, especially CP2 and CP3, increased plant P accumulation and P transfer to grain.

**Table 3:** Effects of different phosphorus treatments on phosphorus accumulation and distribution in different organs of spring maize (2022 and 2023).

Year	Treatment	P accumulation in vegetative organs (kg ha^-1^)			P accumulation of grain (kg ha^-1^)	P accumulation after silking (kg ha^-1^)
		V12	R1	R6	R6	
2022	CP0	19.30 ± 0.14e	55.95 ± 2.71c	31.17 ± 0.58d	64.85 ± 1.20d	40.08 ± 1.57c
	CP1	26.68 ± 1.23d	61.15 ± 1.63b	32.73 ± 0.75c	85.82 ± 1.96c	57.40 ± 1.31b
	CP2	40.68 ± 0.79a	72.80 ± 1.65a	36.81 ± 0.86a	99.62 ± 1.61a	63.62 ± 2.47a
	CP3	38.77 ± 0.25b	70.98 ± 3.68a	36.88 ± 0.65a	97.86 ± 0.84a	63.76 ± 2.83a
	CK	36.39 ± 0.72c	69.23 ± 1.13a	34.76 ± 0.57b	93.49 ± 1.56b	59.01 ± 0.94b
2023	CP0	18.33 ± 0.13d	48.74 ± 2.83d	31.10 ± 0.39c	60.10 ± 0.76d	42.46 ± 1.72c
	CP1	25.64 ± 1.07c	55.59 ± 1.18c	30.99 ± 0.21c	81.26 ± 0.55c	56.66 ± 1.48b
	CP2	37.13 ± 0.54a	67.53 ± 2.42a	36.81 ± 0.54a	96.71 ± 2.98a	65.99 ± 5.21a
	CP3	37.55 ± 0.48a	66.65 ± 1.40ab	37.06 ± 0.47a	94.97 ± 1.38a	65.38 ± 2.41a
	CK	34.75 ± 0.60b	63.50 ± 0.99b	33.65 ± 0.80b	88.82 ± 2.11b	58.97 ± 3.20b
ANOVA						
Year (Y)		**	**	*	**	NS
Treatment(T)		**	**	**	**	**
Y×T		NS	NS	NS	**	NS

CP0, CP1, CP2, CP3, and CK denote no phosphorus application, split phosphorus application through drip irrigation with a 30% reduction, split phosphorus application through drip irrigation with a 15% reduction, split phosphorus application through drip irrigation at the conventional phosphorus rate, and conventional one time basal phosphorus application, respectively. Different letters within a column indicate significant differences among treatments within the same year at the (*p < 0.05*) level. NS indicates no significant difference. Asterisks *, ** denote significance at (*p < 0.05*) and (*p < 0.01*), respectively.

### 3.6 Effects of split phosphorus application through drip irrigation on maize yield

**Table 4** shows that grain yield did not differ significantly between CP2 and CP3. Compared with CP1 and CK, CP2 increased grain yield by 6.79% and 4.07%, respectively (*p < 0.05*), whereas CP3 increased grain yield by 6.08% and 3.38%, respectively (*p < 0.05*). Treatment differences in yield were mainly associated with grain number per ear. Grain number per ear did not differ between CP2 and CP3, but both treatments had higher values than the other treatments. Thus, split P fertigation increased maize yield primarily by increasing grain number per ear.

**Table 4:** Effects of different phosphorus treatments on yield and yield components of spring maize (2022 and 2023).

Year	Treatments	Harvested ears (×10^4^ ears ha^−1^)	Grains per ear	Weight of 1000 grains (g)	Yield (t ha^−1^)
2022	CP0	9.52 ± 0.09a	473.83 ± 4.37d	323.78 ± 3.60b	14.57 ± 0.08c
	CP1	9.54 ± 0.03a	518.86 ± 3.30c	329.92 ± 4.73ab	16.30 ± 0.24b
	CP2	9.60 ± 0.02a	548.72 ± 5.74a	332.38 ± 2.23a	17.45 ± 0.35a
	CP3	9.59 ± 0.02a	549.18 ± 3.13a	330.56 ± 4.69ab	17.40 ± 0.16a
	CK	9.54 ± 0.04a	529.12 ± 2.25b	333.53 ± 2.14a	16.80 ± 0.11b
2023	CP0	9.47 ± 0.06a	471.51 ± 10.54d	321.91 ± 5.21b	14.36 ± 0.23c
	CP1	9.48 ± 0.04a	521.96 ± 2.89c	325.34 ± 3.08ab	16.10 ± 0.17b
	CP2	9.50 ± 0.03a	542.73 ± 2.79a	332.78 ± 4.56a	17.15 ± 0.19a
	CP3	9.50 ± 0.06a	542.16 ± 3.96a	329.99 ± 2.11a	16.97 ± 0.22a
	CK	9.46 ± 0.04a	527.71 ± 2.26b	329.24 ± 4.41ab	16.44 ± 0.28b
ANOVA					
Year (Y)		**	*	NS	**
Treatment (T)		NS	**	**	**
Y×T		NS	*	NS	NS

CP0, CP1, CP2, CP3, and CK denote no phosphorus application, split phosphorus application through drip irrigation with a 30% reduction, split phosphorus application through drip irrigation with a 15% reduction, split phosphorus application through drip irrigation at the conventional phosphorus rate, and conventional one time basal phosphorus application, respectively. Different letters within a column indicate significant differences among treatments within the same year at the (*p < 0.05*) level. NS indicates no significant difference. Asterisks *, ** denote significance at (*p < 0.05*) and (*p < 0.01*), respectively.

### 3.7 Effects of split phosphorus application through drip irrigation on phosphorus use efficiency of maize

**Figure 9** shows that PAE, PRE, and PFPP followed similar trends in both years. Compared with CP1, CP3, and CK, CP2 significantly increased PAE by 34.66%, 22.59%, and 54.62%, respectively (*p < 0.05*). CP2 also increased PRE by 82.84%, 58.31%, and 77.36%, respectively (*p < 0.05*). PFPP was highest under CP1 and exceeded CP2, CP3, and CK by 13.71%, 34.76%, and 39.22%, respectively (*p < 0.05*). These results indicate that CP2 improved P uptake efficiency and agronomic efficiency while maintaining high yield.

**Figure 9:**
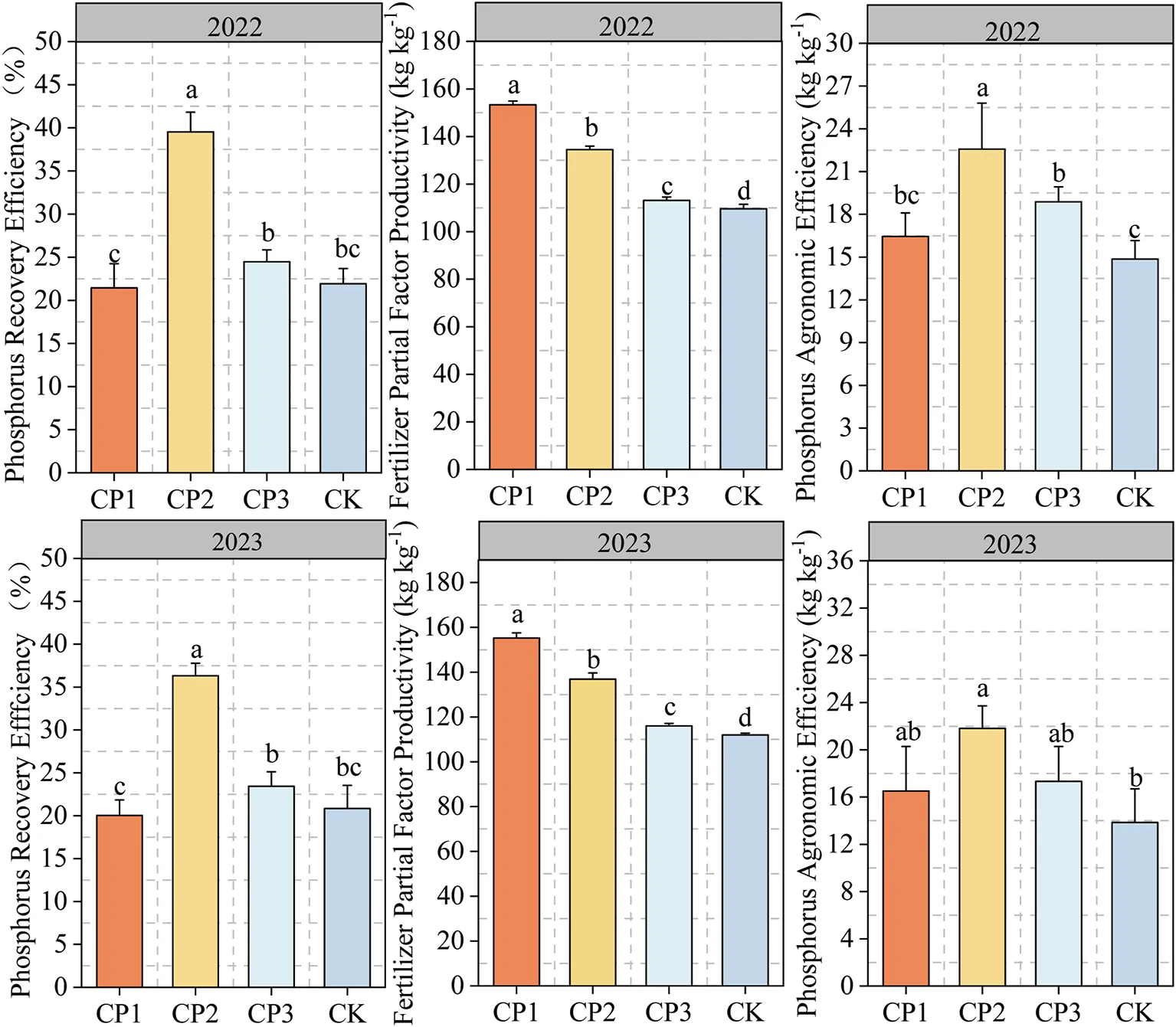
Effects of different phosphorus treatments on apparent phosphorus recovery efficiency (PRE), phosphorus agronomic efficiency (PAE), and partial factor productivity of phosphorus fertilizer (PFPP) in spring maize (2022 and 2023). Different lowercase letters indicate significant differences among treatments at p < 0.05.

### 3.8 Structural equation analysis

The PLS-SEM was constructed to clarify the direct and indirect pathways regulating maize grain yield (**Figure 10**). Phosphate fertilizer application methods significantly affected soil Olsen P availability and root morphological traits (RMTs). Olsen P exerted direct positive effects on RMTs and plant P uptake, whereas plant P uptake directly promoted biomass accumulation and phosphorus use efficiency indicators (PUEIs). Biomass accumulation and PUEIs were the two direct determinants of grain yield, with path coefficients of 0.59 and 0.33, respectively. Phosphate fertilizer application methods, Olsen P, RMTs, and plant P uptake indirectly influenced grain yield through biomass accumulation and PUEIs. These results indicate that optimized P fertigation improved maize yield primarily by enhancing soil P availability, plant P acquisition, biomass formation, and P use efficiency.

**Figure 10:**
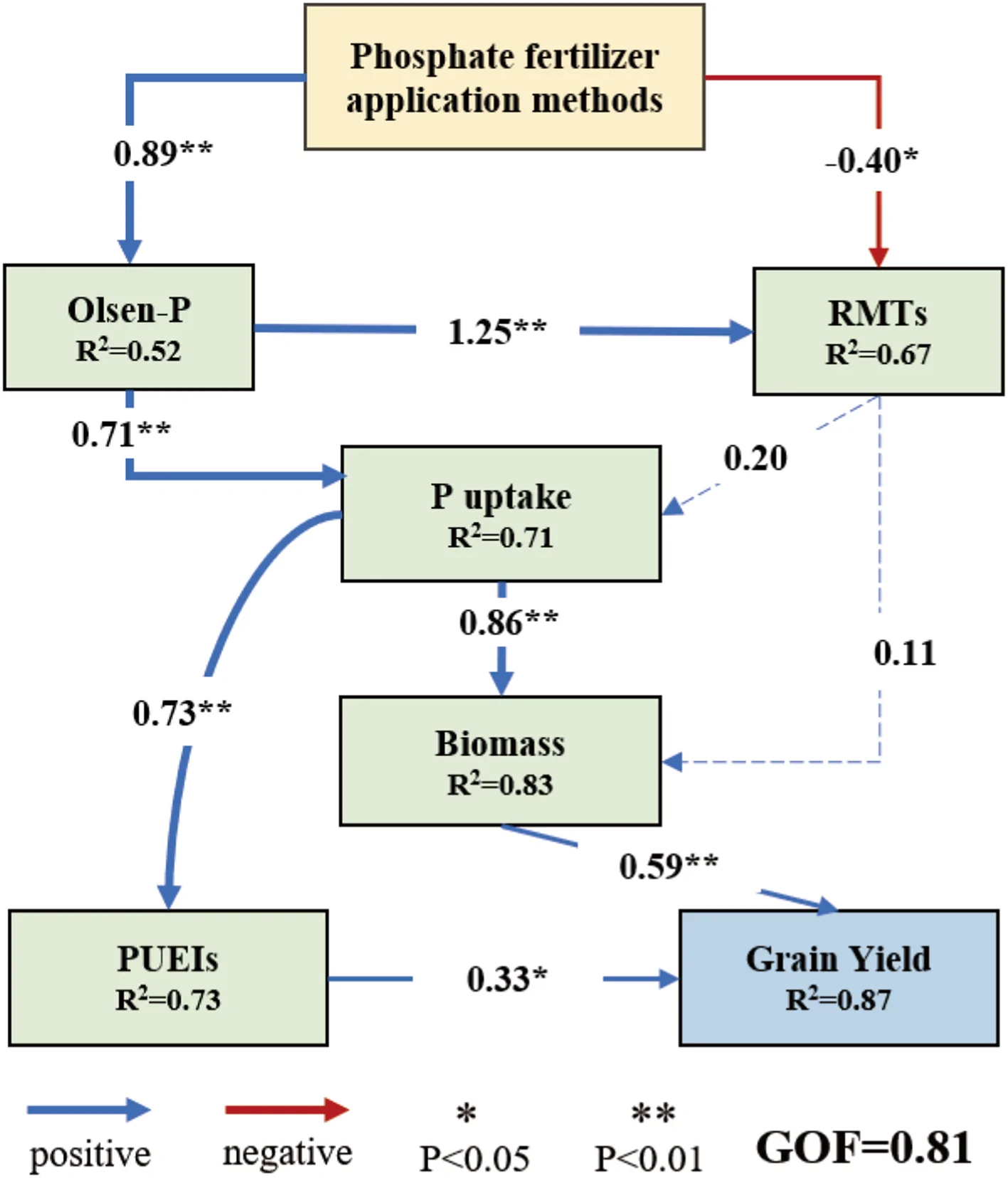
Partial least squares structural equation modeling (PLS-SEM) analysis of the direct and indirect pathways by which phosphate fertilizer application methods affected maize grain yield through soil Olsen P availability, root morphological traits, plant P uptake, biomass accumulation, and phosphorus use efficiency indicators. PLS-SEM was used to quantify the regulatory pathways among phosphate fertilizer application methods, Olsen P, root morphological traits (RMTs), plant P uptake, aboveground biomass, phosphorus use efficiency indicators (PUEIs), and grain yield. Blue and red arrows indicate positive and negative path coefficients, respectively. Solid arrows indicate significant paths, whereas dashed arrows indicate weak or indirect paths retained in the model. Numbers adjacent to arrows denote standardized path coefficients, with * and ** indicating significance at (*p < 0.05*) and (*p < 0.01*), respectively. RMTs include root length density (RLD), root surface area (RSA), root volume (RV), and root dry weight (RDW). P uptake, plant phosphorus uptake; PUEIs, phosphorus use efficiency indicators; PFPP, partial factor productivity of phosphorus; PRE, phosphorus recovery efficiency; PAE, phosphorus agronomic efficiency.

## 4 Discussion

Our results showed that the effects of reducing P input under split fertigation depended on the balance between P supply and its spatial configuration within the root zone. Within the same split MAP fertigation system, further reducing P input in CP1 decreased plant P accumulation, aboveground biomass, and grain yield, whereas increasing P input in CP3 provided no additional yield benefit. In contrast, CP2 reduced P input by 15% while maintaining a grain yield comparable to that of CP3 and achieving relatively high phosphorus recovery efficiency (PRE) and phosphorus agronomic efficiency (PAE). CP2 also maintained relatively high Olsen P and root length density (RLD) in the 20 to 40 cm soil layers, where the two variables showed strong local spatial associations. These results support our hypothesis and further suggest that the agronomic benefit of spatial coordination between roots and available P depends on an adequate level of P supply. When P supply is insufficient, improved spatial configuration cannot fully compensate for the reduction in fertilizer input; once P supply exceeds crop demand, additional P provides little further benefit.

### 4.1 Different P management practices altered the spatial configuration of available P and roots within the root zone

Because P has low mobility in soil, crop P acquisition depends not only on the amount of available P but also on its spatial proximity to active roots (Zhou et al., 2025b). Previous studies have shown that split P application can prolong P availability within the root zone, whereas localized P enrichment can stimulate root proliferation and increase the contact area between roots and soil (Blandino et al., 2022; Tiecher et al., 2023). Thus, the effects of P management on crop P acquisition may involve coordinated changes in both the spatial distribution of available P and root foraging.

Compared with the local conventional practice of basal DAP application at sowing (CK), split MAP fertigation produced a different vertical distribution of Olsen P. Under conventional management, relatively high Olsen P was concentrated mainly in the upper soil layer, whereas CP2 and CP3 maintained greater P availability in the 20–40 cm layers, with the strongest response under CP2 in the 30–40 cm layer (**Figure 3**). This result is consistent with previous studies showing that split P application can maintain P availability during crop growth (Rezaei et al., 2025). The results therefore suggest that split fertigation did not simply increase P supply within the root zone, but also altered the vertical distribution of available P. Compared with one-time basal application, repeated P supply avoided concentrating the entire fertilizer input at sowing and helped maintain P availability within the root zone during the growing season (Xu et al., 2025).

Root distribution also responded to P management. CP2 and CP3 increased root dry weight, root surface area, and root volume, and increased RLD in the 10–40 cm soil layers (**Figure 4** and **5**). In particular, CP2 maintained relatively high root abundance in the middle and deeper soil layers. This is consistent with previous evidence that roots can adjust their spatial distribution in response to localized nutrient availability (Liu et al., 2025). More importantly, our results showed that changes in root distribution and available P were not independent. Under CP2, RLD and Olsen P showed positive spatial associations in both the 20–30 and 30–40 cm layers, and this pattern was consistent across the three replicate plots in both years in the sensitivity analysis. This consistency supports a close spatial correspondence between areas of greater root density and greater P availability (**Figure 7**). In contrast, negative relationships between RLD and Olsen P were observed in several other treatment–soil layer combinations (**Figure 7**). These negative associations should not necessarily be interpreted as spatial mismatch between roots and available P. One possible explanation is that greater root proliferation and P uptake locally depleted labile P in the rhizosphere, resulting in lower Olsen P concentrations in soil zones with higher root density. Thus, the observed RLD-Olsen P relationships may reflect a balance between the spatial distribution of available P and root-induced P depletion, rather than a simple one-directional response of roots to soil P availability.

This spatial correspondence extends previous studies that have largely examined either soil P distribution or root responses separately (Yang et al., 2022). For a poorly mobile nutrient such as P, increasing Olsen P or root abundance alone does not necessarily improve P acquisition. Available P must occur within the soil volume that roots can effectively explore before it can contribute to nutrient uptake. CP2 exhibited relatively greater P availability and root proliferation in the middle and deeper portions of the root zone, accompanied by a closer spatial correspondence between root distribution and soil available P.

### 4.2 P supply and root–P spatial relationships were associated with P acquisition, grain yield, and P use efficiency

The agronomic importance of this spatial coordination was first reflected in plant P acquisition. CP2 and CP3 increased plant P accumulation and aboveground biomass (**Table 3**; **Figure 8**). Previous studies have shown that split P application can improve the temporal synchronization between P supply and crop demand (Kang et al., 2021). Our results further suggest that P acquisition may also depend on the spatial accessibility of available P. When relatively high Olsen P and RLD occurred within the same middle and deeper soil layers, the effective soil volume from which plants could acquire P may have increased.

This process may have been particularly important after silking. Maize continues to acquire P during reproductive growth to support grain formation, whereas insufficient P supply in the later growth stages increases dependence on remobilization of P accumulated in vegetative organs before silking (Zhang et al., 2022; Wang et al., 2024b). In our study, CP2 and CP3 had greater P accumulation after silking, while CP2 simultaneously maintained relatively high RLD and Olsen P in the 20–40 cm root zone. The coexistence of active roots and available P in the middle and deeper soil layers may therefore have helped sustain P acquisition during reproductive growth and supported continued dry matter accumulation and grain formation (Zhang et al., 2024).

Yield components provided further evidence that this process may have contributed to grain formation. The yield advantages of CP2 and CP3 were mainly associated with greater grain number per ear, whereas harvested ear number differed little among treatments (**Table 4**). This suggests that optimized P management increased yield primarily through improved grain formation rather than through changes in stand density, possibly because improved plant P nutrition supported reproductive development (Zhang et al., 2018; Yan et al., 2024).

Comparisons among P rates further showed that both the amount of P supplied and its spatial accessibility determined whether these benefits were translated into crop productivity. CP1 also received split fertigation, but plant P accumulation, biomass, and grain yield were lower than those under CP2 and CP3. This indicates that when P input was reduced to the CP1 level, improved timing and placement of fertilizer were insufficient to compensate for the lower total P supply. In contrast, increasing P input in CP3 to the conventional rate did not further increase grain yield, suggesting that P supply was no longer a major constraint above the CP2 level (Jagdeep-Singh and Brar, 2022; Jagdeep-Singh et al., 2023). Thus, CP1 was primarily constrained by insufficient P supply, whereas CP3 showed diminishing returns to additional P input. CP2 achieved a more favorable balance between P supply and its spatial accessibility to roots.

Under split fertigation, crop P acquisition may therefore be constrained by two factors: the amount of available P within the root zone and the extent to which this P is accessible to active roots. When P input is too low, insufficient supply becomes the dominant constraint. Once P supply is adequate, further fertilizer input provides little additional benefit if it does not improve the effective accessibility of P to roots (Yan et al., 2022; Luo et al., 2024). Therefore, the objective of optimized P management should not be to maximize soil P availability, but to provide sufficient P while improving its spatial accessibility within the active root zone.

Changes in P use efficiency were consistent with this interpretation. CP1 had the highest partial factor productivity of P fertilizer (PFPP) because of its lower P input, but its lower P accumulation and grain yield showed that high PFPP alone did not represent the best agronomic performance. CP3 maintained high yield, but additional P input did not provide a further yield benefit, and its PRE and PAE were lower than those of CP2. In contrast, CP2 reduced P input by 15% and increased PRE and PAE by 77.36% and 54.62%, respectively, relative to CK (**Figure 9**). These results indicate that the appropriate P rate cannot be determined from a single efficiency index and should instead consider P supply, plant P acquisition, and grain yield together (Gu et al., 2023).

The PLS-SEM further supported the overall pathway from P conditions in the root zone to crop productivity (**Figure 10**). P management affected Olsen P and root morphological traits, which were subsequently associated with plant P uptake. Greater P uptake promoted biomass accumulation and P use efficiency, which were in turn associated with grain yield. These relationships suggest that the advantage of optimized P management resulted from coordinated changes in soil P availability, root development, plant P acquisition, biomass formation, and P use efficiency (Gong et al., 2024).

Overall, previous studies have separately emphasized the importance of split P application for maintaining P availability and root plasticity for improving P acquisition. Our results further show that their agronomic effects are jointly regulated by the amount of P supplied and its spatial accessibility to roots. When P input was too low, insufficient P supply constrained crop P acquisition; when P input was excessive, additional P was not translated into further yield gains. In contrast, the moderate P reduction in CP2 provided sufficient P while maintaining better spatial coordination between available P and roots in the middle and deeper soil layers, thereby sustaining P acquisition and grain yield and improving P use efficiency. Therefore, achieving lower P input without yield loss under split fertigation depends not simply on reducing fertilizer use, but on improving the spatial accessibility of an adequate P supply to active roots.

This study has three key limitations requiring further research. The two-year trial was conducted at a single site in Inner Mongolia under sandy loam conditions, which may not fully capture interannual climatic variability or the responses under different soil and environmental conditions. Therefore, validation over longer periods and across multiple sites and soil types is needed to assess the broader applicability of these findings. Split MAP fertigation and basal DAP application differed simultaneously in fertilizer source, application method, timing, and placement; therefore, their independent effects could not be separated in the present study, and factorial experiments are needed to distinguish their individual and interactive effects. Third, soil moisture was not continuously monitored in the present study. Because soil water dynamics under drip fertigation can influence both P movement and root distribution, the absence of spatially and temporally resolved soil moisture measurements limits our ability to distinguish the respective contributions of water availability and P availability to the observed root–P spatial patterns. Future studies should therefore integrate simultaneous measurements of soil moisture, available P, and root distribution across space and time. In addition, although CP2 reduced P input while maintaining grain yield and improving P-use efficiency, the present study did not quantify labor, energy, equipment operation, or other costs associated with repeated fertigation events. Therefore, the economic feasibility and profitability of this management strategy cannot be determined from the present data and should be evaluated in future cost–benefit studies. In addition, root spatial distribution was assessed using one representative plant from each replicate plot at R1, which provided detailed spatial information but limited the sampling intensity and did not capture temporal changes in root–soil P relationships across phenological stages.

## 5 Conclusion

This field experiment conducted over two years showed that the effectiveness of P reduction under split fertigation depended on the balance between P supply and its spatial accessibility to maize roots. Within the same split MAP fertigation system, reducing P input to 105 kg P_2_O_5_ ha^-1^ constrained plant P accumulation, biomass production, and grain yield, whereas increasing P input from 127.5 to 150 kg P_2_O_5_ ha^-1^ provided no additional yield benefit. In contrast, the moderate P rate of 127.5 kg P_2_O_5_ ha^-1^ maintained greater Olsen P availability and root proliferation in the middle and deeper root zone, where root length density and Olsen P showed closer spatial correspondence. This spatial coordination was accompanied by greater P acquisition, including P accumulation after silking, and supported biomass production and grain formation. Consequently, CP2 maintained grain yield comparable to the full P rate while reducing P input by 15% and improving P fertilizer use efficiency. These findings indicate that reducing P input without yield loss requires not simply lowering the fertilizer rate, but maintaining sufficient P supply while improving the spatial accessibility of available P to active roots. Under the conditions of this study, 127.5 kg P_2_O_5_ ha^-1^ under split MAP fertigation provided the most favorable balance between P supply, crop P acquisition, grain yield, and P use efficiency.

## Data Availability

The raw data supporting the conclusions of this article will be made available by the authors, without undue reservation.
