# Supplementary material for: Moderate phosphorus reduction under split drip fertigation maintains maize yield and coincides with closer root–available phosphorus spatial correspondence
Source: Front Plant Sci. 2026 Oct 13;17:1999323. doi: 10.3389/fpls.2026.1999323 (PMC13710221; doi:10.3389/fpls.2026.1999323)
Supplement: Supplementary file 1 [file Table1.docx]

Supplementary material

Supplementary Table S1. Effects of different phosphorus treatments on soil Olsen P at different soil depths in 2022 and 2023.

| Year | Treatment | Layer（cm） | | | |
| --- | --- | --- | --- | --- | --- |
|  |  | 0-10 | 10-20 | 20-30 | 30-40 |
| 2022 | CP0 | 18.72 ± 0.68d | 14.96 ± 0.36c | 6.69 ± 0.86d | 4.95 ± 0.73c |
|  | CP1 | 20.80 ± 0.82c | 17.04 ± 0.74bc | 13.99 ± 0.56c | 5.97 ± 1.03c |
|  | CP2 | 27.60 ± 0.47a | 16.47 ± 1.40bc | 16.00 ± 0.65b | 12.00 ± 0.40a |
|  | CP3 | 27.04 ± 0.80a | 17.93 ± 0.52b | 18.24 ± 0.82a | 9.05 ± 0.73b |
|  | CK | 25.38 ± 2.16a | 24.37 ± 1.65a | 13.18 ± 0.77c | 8.23 ± 0.55b |
| 2023 | CP0 | 19.16 ± 1.18d | 15.91 ± 0.16c | 7.45 ± 0.48d | 5.48 ± 0.44d |
|  | CP1 | 22.39 ± 0.98c | 17.58 ± 0.98b | 14.66 ± 0.62c | 6.67 ± 1.25cd |
|  | CP2 | 29.97 ± 0.74a | 17.17 ± 1.59b | 17.28 ± 0.25b | 12.84 ± 1.30a |
|  | CP3 | 27.45 ± 1.69a | 18.20 ± 1.00b | 19.28 ± 0.99a | 9.39 ± 0.80b |
|  | CK | 28.02 ± 0.68a | 25.76 ± 0.97a | 14.70 ± 0.66c | 8.40 ± 0.90bc |
| ANOVA |  |  |  |  |  |
| Year(Y) |  | ** | ns | ** | ns |
| Treatment(T) |  | ** | * | ** | ** |
| Y×T |  | ns | ns | ns | ns |

Supplementary Figure S1. Grid used for root sampling in three dimensions in the field.
